# 11-beta-hydroxysteroid dehydrogenase type 1 (*HSD11B1*) gene expression in muscle is linked to reduced skeletal muscle index in sarcopenic patients

**DOI:** 10.1007/s40520-023-02574-w

**Published:** 2023-11-09

**Authors:** Sabine Schluessel, Wei Zhang, Hanna Nowotny, Martin Bidlingmaier, Stefan Hintze, Sonja Kunz, Sebastian Martini, Stefan Mehaffey, Peter Meinke, Carl Neuerburg, Ralf Schmidmaier, Benedikt Schoser, Nicole Reisch, Michael Drey

**Affiliations:** 1 Department of Medicine IV, University Hospital, Ludwig Maximilian University Munich, Ziemssenstraße 5, 80336 Munich, Germany; 2 Department of Neurology, Friedrich-Baur-Institute, LMU Klinikum, LMU Munich, Munich, Germany; 3 Department of Orthopaedics and Trauma Surgery, Musculoskeletal University Center Munich (MUM), University Hospital, LMU Munich, Munich, Germany

**Keywords:** Glucocorticoids, Sarcopenia, *NR3C1*, *HSD11B1*, *FBXO32*, *TRIM63*

## Abstract

**Background:**

Glucocorticoids play a significant role in metabolic processes and pathways that impact muscle size, mass, and function. The expression of 11-beta-hydroxysteroid dehydrogenase type 1 (HSD11B1) has been previously described as a major regulator of skeletal muscle function in glucocorticoid-induced muscle atrophy and aging humans. Our study aimed to investigate glucocorticoid metabolism, including the expression of HSD11B1 in skeletal muscle, in patients with sarcopenia.

**Methods:**

Muscle biopsies were taken from the vastus lateralis muscle of thirty-three patients over 60 years of age with hip fractures. Sarcopenia status was assessed according to the criteria of the European Working Group on Sarcopenia in Older People 2. Skeletal muscle mass was measured by bioelectrical impedance analysis. Cortisol and cortisone concentrations were measured in serum. Gene expression analysis of *HSD11B1, NR3C1*, *FBXO32,* and *TRIM63* in muscle biopsies was performed. Serial cross sections of skeletal muscle were labeled with myosin heavy chain slow (fiber type-1) and fast (fiber type-2) antibodies.

**Results:**

The study included 33 patients (21 women) with a mean age of 82.5 ± 6.3 years, 17 patients revealed sarcopenic (*n* = 16 non-sarcopenic). Serum cortisone concentrations were negatively correlated with muscle mass (*ß* =  − 0.425; *p* = 0.034) and type-2 fiber diameter (*ß* =  − 0.591; *p* = 0.003). Gene expression of *HSD11B1* (*ß* =  − 0.673; *p* = 0.008) showed a negative correlation with muscle mass in the sarcopenic group. A significant correlation was found for the non-sarcopenic group for *NR3C1* (*ß* = 0.548; *p* = 0.028) and muscle mass.

**Conclusion:**

These findings suggest a pathogenetic role of *HSD11B1* in sarcopenic muscle.

## Background

The progressive age-related decrease in skeletal muscle mass and strength is termed sarcopenia and affects mortality, independence and quality of life in older adults [1]. Multiple endocrinological changes, e.g., decrease of androgens and growth factors as well as glucocorticoid (GC) dysregulation, play an essential role in the pathophysiology of sarcopenia [2–4]. GC-induced muscle atrophy and hypercortisolism are similar to sarcopenia in that they are all characterized by decreased muscle strength and mass [5, 6]. Therefore, several studies have investigated the relationship between serum concentrations of GCs and sarcopenia, showing a general increase in mean daily cortisol levels in older patients [3, 7–9].

GCs are vital in the body's stress response system regulated by the hypothalamic–pituitary–adrenal axis [7]. The cortisol concentrations show a distinct circadian rhythm, with the highest concentrations in the morning and lowest in the evening [10]. GCs have catabolic effects on muscle tissue, breaking down muscle protein for energy [11]. In the short term, this can be beneficial, allowing quick access to energy stores during stress or physical activity [7]. However, chronically elevated cortisol levels cause muscle wasting and loss of muscle mass, as highlighted in patients with GC excess, Cushing’s syndrome [12].

Studies on GC-induced muscle atrophy show that GCs stimulate protein breakdown and inhibit protein synthesis in skeletal muscle, resulting in muscle mass loss [5]. Skeletal muscle consists of two types of muscle fibers, slow twitch (type 1) and fast twitch (type 2), and GCs can affect both types. However, type-2 fibers, responsible for dynamic power and speed generation, are more susceptible to GC catabolic effects than the more static working type-1 fibers [5]. As a result, individuals taking prescribed GCs may experience more pronounced muscle weakness and atrophy in type-2 muscle fibers, leading to a decline in overall physical function [13, 14]. This loss of muscle mass, particularly in fast-twitch type-2 muscle fibers, can shift muscle fiber composition toward a higher proportion of slow-twitch type-1 fibers, which are more resistant to GC-induced atrophy [13].

On a molecular level, the enzyme 11-beta-hydroxysteroid dehydrogenase type 1 (HSD11B1) regulates cortisol availability within cells [15]. It is expressed in skeletal muscle and converts inactive cortisone to active cortisol [16]. The binding of cortisol to its receptor, nuclear receptor subfamily 3, group C, member 1 (NR3C1), induces a conformational change in NR3C1, causing it to translocate from the cytoplasm to the nucleus. It binds to specific DNA sequences known as glucocorticoid response elements (GREs) [17]. This binding leads to changes in gene expression and causes transcription of F-box only protein 32 (FBXO32), also called Atrogin-1, and E3 ubiquitin-protein ligase TRIM63, also known as Muscle Ring-Finger Protein-1 (MuRF-1) [6]. FBXO32 and TRIM63 lead to myofibril degradation and thereby cause GC-induced muscle atrophy [6]. FBXO32 and TRIM63 are referred to as atrogene markers.

Similar to GC-induced muscle atrophy, increased serum GC levels and decreased type-2 fiber diameters have also been reported in sarcopenia [5, 18]. Therefore, our study aimed to investigate the GC pathway in human sarcopenic muscle cells and compare it to an age-matched control group. Specifically, we intended to histologically characterize the fiber types, determine cortisol/cortisone serum levels, and analyze the quantitative gene expression of *NR3C1*, *HSD11B1*, *FBXO32,* and *TRIM63* in muscle tissue and correlate them to the clinical phenotype of sarcopenic and non-sarcopenic patients.

## Methods

### Participants

In collaboration with the Department of General-, Trauma- and Reconstructive Surgery at the University Hospital Munich, we recruited 33 patients aged 60 years or older who underwent surgery with hip fracture between November 2017 and March 2019. Exclusion criteria included age younger than 60 years, specific neuromuscular diseases (such as myasthenia gravis, muscular dystrophy, amyotrophic lateral sclerosis, polio, myositis), dementia, chronic inflammatory diseases (such as Crohn’s disease, ulcerative colitis, rheumatoid arthritis), GC therapy, and cancer therapy in the last five years. The study protocol was approved by the ethics committee of the medical faculty of the LMU Munich (IRB-No. 328–15). All participants included in the study provided written informed consent before surgery.

### Study groups

This study consisted of two groups: the sarcopenic group, which comprised all patients meeting the criteria for sarcopenia (as per the sarcopenia assessment), and a control group referred to as all probands who did not meet the criteria for sarcopenia.

### Sarcopenia assessment

Sarcopenia was defined according to the criteria of the European Working Group on Sarcopenia in Older People 2 (EWGSOP2) [1]. Bioelectrical impedance analysis (BIA) was performed under standard conditions, including a supine position and surface electrodes on the wrist and ankle of the non-fractured side. Appendicular lean mass (aLM) was estimated using the equation developed by Sergi et al. [19]. The skeletal muscle index (SMI) was calculated by dividing aLM by squared body height. Low muscle mass was defined as SMI below 7.0 kg/m^2^ in men and 5.5 kg/m^2^ in women [1]. Handgrip strength was measured with a Saehan DHD-1 Digital Hand Dynamometer in an upright sitting position with the arm held in 90-degree flexion. Three consecutive measurements of both hands were taken, and the maximum value was used. Thresholds for handgrip strength were 27 kg for men and 16 kg for women [1]. The combination of low muscle mass and low handgrip strength defines the diagnosis of sarcopenia. Pre-sarcopenia is already present if handgrip strength is below the thresholds. Due to a small number of cases, pre-sarcopenia and sarcopenia were combined to one cohort in our study. A z-score combining handgrip strength and muscle mass was calculated separately for men [z-score sarcopenia men = [(27 – handgrip strength)/SD (handgrip strength)] + [(7.0 – SMI)/SD (SMI)] and women [z-score sarcopenia women = [(16 – handgrip strength)/SD (handgrip strength)] + [(5.5 – SMI)/SD (SMI)]. The higher the z-score, the more sarcopenic the patient. Measurements were conducted between two and seven days after surgery, with a mean value of four days.

### Muscle biopsies

Open biopsies of the musculus vastus lateralis were performed during hip fracture surgery. Muscle samples were taken at the attachment of the vastus lateralis muscle near the greater trochanter. The biopsies were directly cryo-conserved. Muscle tissue blocks were cut to 10 μm tissue slices on a cryostat (HM505E; Micron, Walldorf, Germany) at − 30° C and mounted on glass slides (double frosted microscope slides; Fisher Scientific), air dried for 2 h, and stored at − 80 °C.

### Immunohistochemistry and fiber size measurement

Muscle sections were taken from − 80 °C storage and left to air dry for 15 min at room temperature. To hydrate the sections, 1 × TBS was used for three washes at a 5-min interval. The sections were then blocked for 1 h at room temperature using a solution comprising 1 × TBS, 0.1% tween, and 0.9% cold water fish gelatine. Primary antibodies were then added and incubated overnight. After incubation, the sections underwent several washes using 1 × TBS and 0.1% tween. Finally, secondary antibodies were added and incubated for 1 h. After two final washes, the samples were covered with mounting medium and a coverslip. The following primary antibodies and dilutions were used for immunohistochemistry: anti-MHC slow (M8421, Sigma-Aldrich: 1:3000) and anti-MHC fast (M4276, Sigma-Aldrich: 1:3000). The secondary antibodies utilized were Discovery™ Universal Secondary antibodies for use with Discovery™ Detection kits on the Ventana Discovery™ Staining Platform. Pictures of fiber staining were obtained using an Olympus CKX53 microscope equipped with a UC90 camera. The fiber sizes were measured using the cellSens Software (Olympus) on these pictures. To avoid distortion that may occur when a muscle fiber is cut obliquely, the "lesser diameter" was measured [20]. Each biopsy had an average of 250 muscle fibers evaluated.

### cDNA synthesis and qPCR analysis

Ribonucleic acid (RNA) was extracted from cryopreserved cells and transcribed into complementary Deoxyribonucleic acid (cDNA) by reverse transcription using the SuperScript II kit (Thermofisher, Waltham, MA, US). Using Taqman assay (Taqman Fast Advanced Master Mix, Thermofisher, Waltham, MA, US), the relative gene expression of *NR3C1*, *HSD11B1*, *FBXO32,* and *TRIM63* (all primers Thermofisher, Waltham, MA, US) was analyzed in Lightcycler 2.0 (Roche Diagnostic, Mannheim, Germany). The relative expression for each gene was calculated using the 2^–∆∆Ct^ method using *18srRNA* (Thermofisher, Waltham, MA, US) as a reference gene [21].

### Western Blot analysis

Proteins were extracted from 40 × 10 µm muscle sections (*n* = 18, nine control and nice sarcopenic samples) using RIPA (Radioimmunoprecipitation Assay) buffer with protease inhibitors (cOmplete Ultra® Roche #05892970001) and an ultrasonic sonicator with a MS73 tip (Bandelin Sonopuls) to lyse the sections. SDS PAGE was used to separate the proteins on a 4%-15% TGX gel (BioRad #4,568,083). The proteins were transferred via semi dry Western blot (Trans-Blot® Turbo™ system by BioRad) onto nitrocellulose membranes (Trans-Blot® Turbo™ RTA Transfer Kit #1,704,270). For blocking we used the Intercept® Blocking Buffer (LI-COR #927–60,001). GAPDH (Merck Millipore #MAB374) was used for normalization and the anti-h/m/r11β-HSD1 (R&D Systems #AF3397) was used to detect HSD11B1. As secondary antibodies we used donkey anti-mouse IRDye 680RD (LI-COR# 926-68072) and donkey anti-rabbit IRDye 800 CW (Li-COR #926-32214). All images were obtained using a LI-COR FC imaging system. For quantification we used LI-CORs I, magingStudio software. All Western blots were repeated at least three times to confirm the results.

### Laboratory measurements

Blood samples for measurement of serum concentrations were obtained on the third postoperative day. After centrifugation, the serum was stored at − 80° Celsius until analysis. Serum hormone concentrations (ng/ml) of cortisol and cortisone were measured at the endocrine laboratory of Department of Medicine IV of the University Hospital Munich (LMU, Germany) by liquid chromatography-tandem mass spectrometry (LC-MS/MS). Chromatography was performed on the 1290 Infinity II HPLC System (Agilent Technologies, Santa Clara, CA, USA) coupled to a QTrap 6500 + tandem mass spectrometer (Sciex, Framingham, MA, USA). Validation data for all assays and reference intervals have been published [22].

### Statistical analysis

Statistical analyses were performed using IBM software SPSS v29.0 (IBM-SPSS Inc., Chicago, II, USA). Participants’ characteristics are expressed as mean values and their standard deviation. Group differences were calculated by Student’s *t*-test. All correlations are shown as Pearson’s coefficient. Statistical significance was set at *p* < 0.05 for all analyses.

## Results

The characteristics of the participants are displayed in Table 1. Thirty three patients were enrolled in the study, 21 women and 12 men, with a mean age of 82.5 ± 6.3 years. The groups were categorized based on sex. The only significant difference was observed in the severity of sarcopenia measured by the z-score sarcopenia (*p* = 0.014), indicating that males were more impacted by sarcopenia than females. However, no significant differences existed between males and females for any other characteristics.

**Table 1:** Patients’ characteristics

	All (*n*=33)	Men (*n*=12)	Women (*n*=21)	*P value*
Clinical characteristics				
Age (years)	82.5 (6.3)	83.8 (5.3)	81.8 (6.8)	0.400
BMI (kg/m²)	24.0 (4.1)	23.5 (4.3)	24.3 (4.0)	0.612
Markers for sarcopenia				
Handgrip strength (kg)	21.5 (7.7)	24.7 (8.3)	19.6 (6.8)	0.065
SMI (kg/m²)	6.9 (1.2)	7.3 (1.2)	6.6 (1.1)	0.103
Z-score sarcopenia	−1.0 (1.7)	-0.0 (1.8)	−1.5 (1.4)	**0.014**
Fiber type-1 diameter (µm)	63.0 (17.3)	60.7 (13.0)	64.3 (19.7)	0.597
Fiber type-2 diameter (µm)	44.9 (9.9)	49.3 (11.8)	43.5 (7.8)	0.107
Laboratory measurements				
Cortisol (ng/ml)	151.8 (50.5)	143.1 (46.2)	155.8 (53.3)	0.569
Cortisone (ng/ml)	17.5 (4.4)	15.3 (2.5)	18.6 (4.7)	0.080

All measures are presented as mean ± SD Abbreviations: *BMI* Body mass index, *SMI* Skeletal muscle index; *p* Values <.05 are bold; eight probands were missing laboratory measurements

^a^Student’s *t*-test, ^b^Chi-square-test

## Clinical markers for sarcopenia and serum levels of cortisone and cortisol

**Fig. 1:**
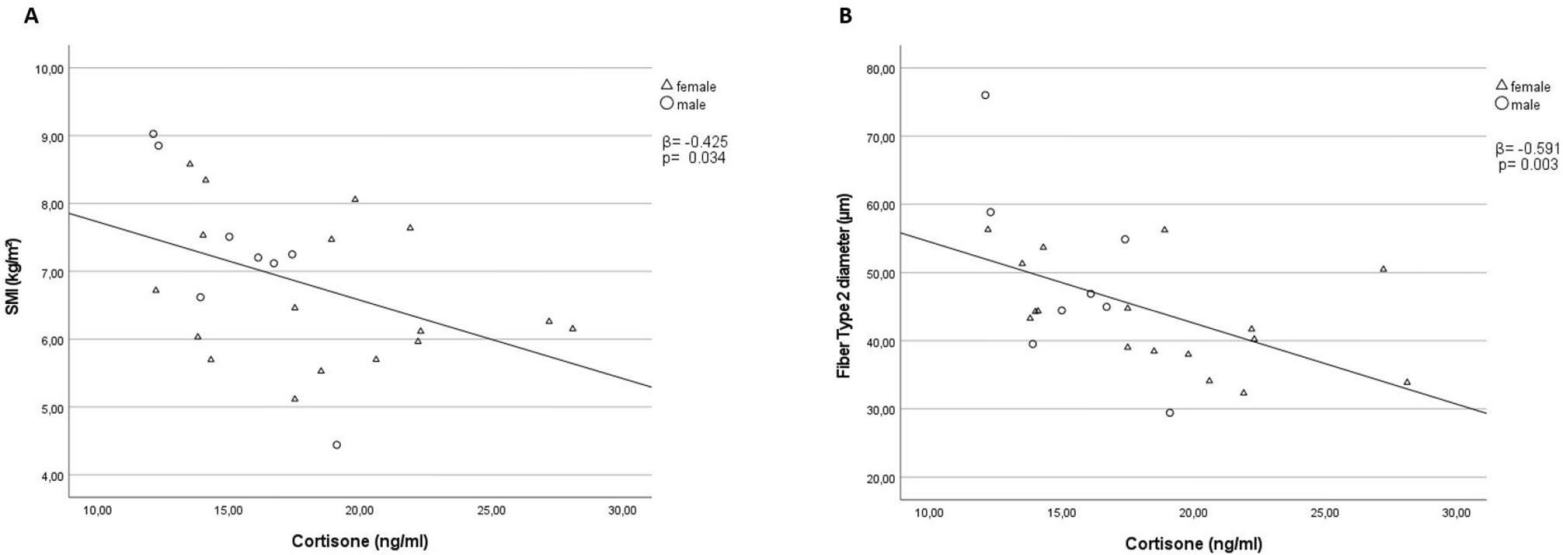
Correlation between SMI, type-2 fiber diameter and cortisone.Pearson’s correlations for all probands between cortisone serum levels and SMI (**A**); Fiber type-2 diameter (**B**); Abbreviations: *SMI* Skeletal muscle index

Figure 1a and Table 2 present the relationship between muscle mass (measured by SMI) and cortisone serum concentrations. Pearson's correlation coefficient of − 0.425 (*p* = 0.035) indicates that patients with higher SMI exhibit lower serum cortisone levels. Similarly, in Fig. 1b and Table 2, the relationship between type-2 fiber diameter and cortisone serum concentrations is shown. The Pearson's correlation coefficient of − 0.591 (*p* = 0.003) presents those patients with larger type-2 fiber diameters displaying lower serum cortisone levels. No significant results were found for cortisol serum levels (Table 2).

**Table 2:** Correlations between markers of sarcopenia, gene expression (NR3C1, HSD11B1, FBXO32, TRIM63) and serum levels (cortisol, cortisone)

Markers for sarcopenia	All						Sarcopenic group						Control group					
	NR3C1	HSD11B1	FBXO32	TRIM63	Cortisol	Cortisone	NR3C1	HSD11B1	FBXO32	TRIM63	Cortisol	Cortisone	NR3C1	HSD11B1	FBXO32	TRIM63	Cortisol	Cortisone
Handgrip strength																		
Correlation																		
Coefficient	0,073	−0,044	*–*0,060	0,194	*–*0,175	*–*0,131	*–*0,048	*–*0,320	*–*0,016	*–*0,163	*–*0,288	*–*0,223	0,511^*^	*–*0,042	0,212	0,397	*–*0,114	*–*0,440
* p value*	0,687	0,819	0,742	0,280	0,402	0,531	0,854	0,265	0,952	0,531	0,391	0,509	0,043	0,877	0,430	0,128	0,697	0,115
SMI																		
Correlation																		
Coefficient	0,374^*^	*–*0,183	0,133	0,074	*–*0,140	*–*0,425^*^	0,329	*–*0,673^**^	0,142	*–*0,059	*–*0,404	*–*0,380	0,548^*^	0,299	0,138	0,217	*–*0,010	*–*0,555^*^
* p value*	0,032	0,334	0,460	0,682	0,506	0,034	0,198	0,008	0,587	0,822	0,218	0,249	0,028	0,261	0,610	0,420	0,974	0,039
Z*-*score sarcopenia																		
Correlation																		
Coefficient	*–*0,181	0,109	0,046	*–*0,128	0,073	0,031	*–*0,332	0,767^**^	*–*0,222	*–*0,352	0,174	0,343	*–*0,228	*–*0,296	0,243	0,188	*–*0,068	0,244
* p value*	0,314	0,566	0,801	0,477	0,728	0,883	0,193	0,001	0,393	0,166	0,610	0,302	0,396	0,266	0,365	0,486	0,818	0,402
Fiber type-1																		
diameter																		
Correlation																		
Coefficient	0,062	0,166	0,134	0,014	0,207	0,224	0,056	*–*0,021	0,053	0,156	0,055	*–*0,711^*^	0,117	0,245	0,456	0,098	0,267	0,265
* p value*	0,746	0,389	0,481	0,943	0,332	0,292	0,849	0,945	0,858	0,594	0,880	0,021	0,678	0,378	0,088	0,729	0,378	0,382
Fiber type-2																		
diameter																		
Correlation																		
Coefficient	0,425^*^	*–*0,263	0,281	0,134	*–*0,048	*–*0,591^**^	0,578^*^	*–*0,474	0,313	0,340	*–*0,203	*–*0,590	0,409	*–*0,167	0,549^*^	0,249	0,013	*–*0,675^*^
* p value*	0,021	0,175	0,140	0,489	0,828	0,003	0,030	0,102	0,276	0,235	0,574	0,072	0,146	0,567	0,042	0,390	0,967	0,016

All correlations are shown as Pearson’s coefficient, *p* values <0.05 are highlighted. Abbreviations: *SMI* Skeletal muscle index, *NR3C1* Glucocorticoid Receptor, *HSD11B1* Hydroxysteroid 11-Beta Dehydrogenase 1, *FBXO32* F-box only protein 32 (Atrogin-1), *TRIM63 MuRF-1* (Muscle Ring-Finger Protein-1)

## Clinical markers for sarcopenia and *HSD11B1*/*NR3C1* gene expression in muscle biopsies

**Fig. 2:**
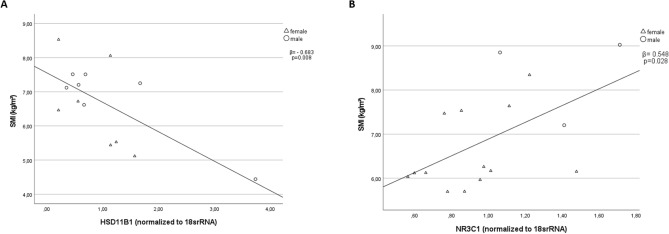
Correlations between SMI and gene expression profiles: Pearson’s correlations between SMI and HSD11B1(**a**) in the sarcopenic group and between SMI and NR3C1 (**b**) in the control group; Abbreviations: *SMI* Skeletal muscle index, *HSD11B1* Hydroxysteroid 11-Beta Dehydrogenase 1, *NR3C1* Glucocorticoid Receptor

No significant differences were found in the gene expression profiles of *HSD11B1*, *NR3C1*, *FBXO32*, and *TRIM63* between the sarcopenic and control groups (data not shown). However, a significant negative correlation was observed between SMI and *HSD11B1* in the sarcopenic group (*β* =  − 0.673, *p* = 0.008, as depicted in Fig. 2a and Table 2*)*. Additionally, a significant positive correlation was found between SMI and *NR3C1* in the control group (*β* = 0.548, *p* = 0.028; Fig. 2b and Table 2). This positive correlation was insignificant in the sarcopenic group (*β* = 0.329, *p* = 0.198; Fig. 2b and Table 2).

## Skeletal muscle atrogenes and *NR3C1* gene expression

**Fig. 3:**
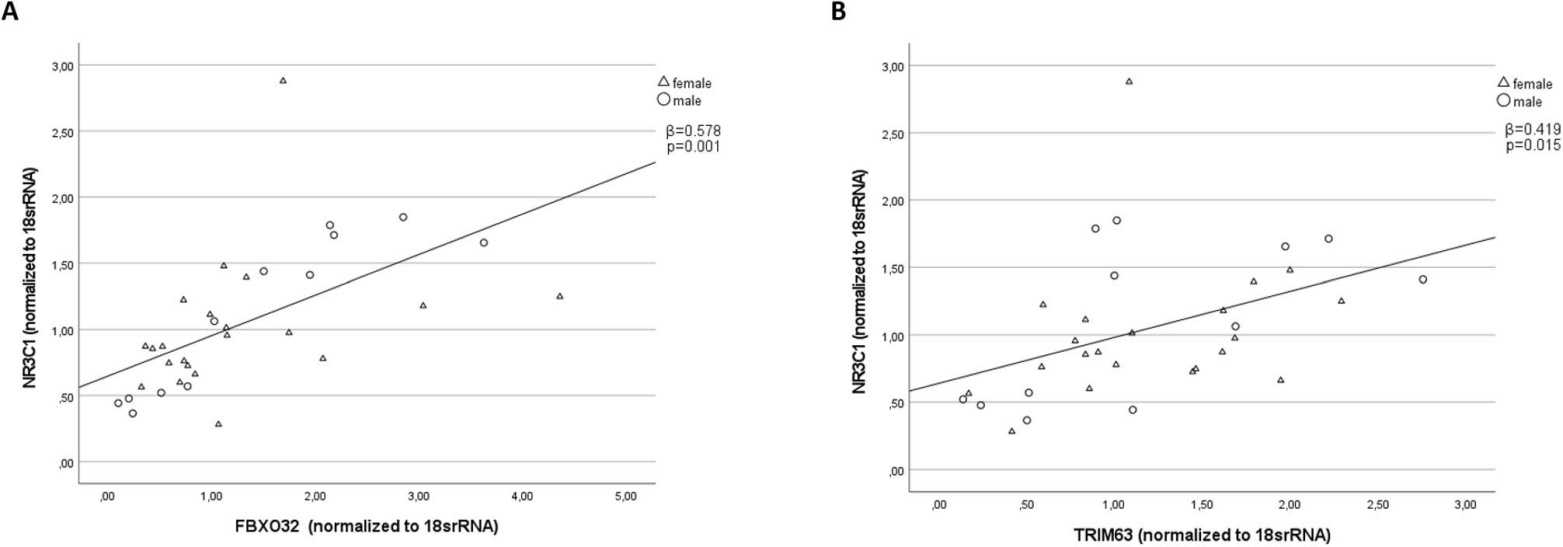
Correlation between NR3C1 and atrogenes (FBXO32 and TRIM63). Pearson’s correlations for all probands between NR3C1 and FBXO32 (**A**); TRIM63 (**B**); Abbreviations: *NR3C1* Glucocorticoid Receptor; *FBXO32* F-box protein 32; *TRIM63* tripartite motif containing 63

Figure 3 illustrates the findings regarding the gene expressions of two muscle atrophy-related genes, FBXO32 (also known as Atrogin-1; *β* = 0.578, *p* = 0.001) and TRIM63 (also referred to as MuRF1; *β* = 0.419, *p* = 0.015), in skeletal muscle of all probands. Our analysis reveals a positive correlation between the expression levels of these two atrophy-related genes and NR3C1.

Figure 3 illustrates that the gene expressions of FBXO32 (*β* = 0.578, *p* = 0.001) and TRIM63 (*β* = 0.419, *p* = 0.015) positively correlate with NR3C1 in skeletal muscle.

## HSD11B1 on protein levels

**Fig. 4:**
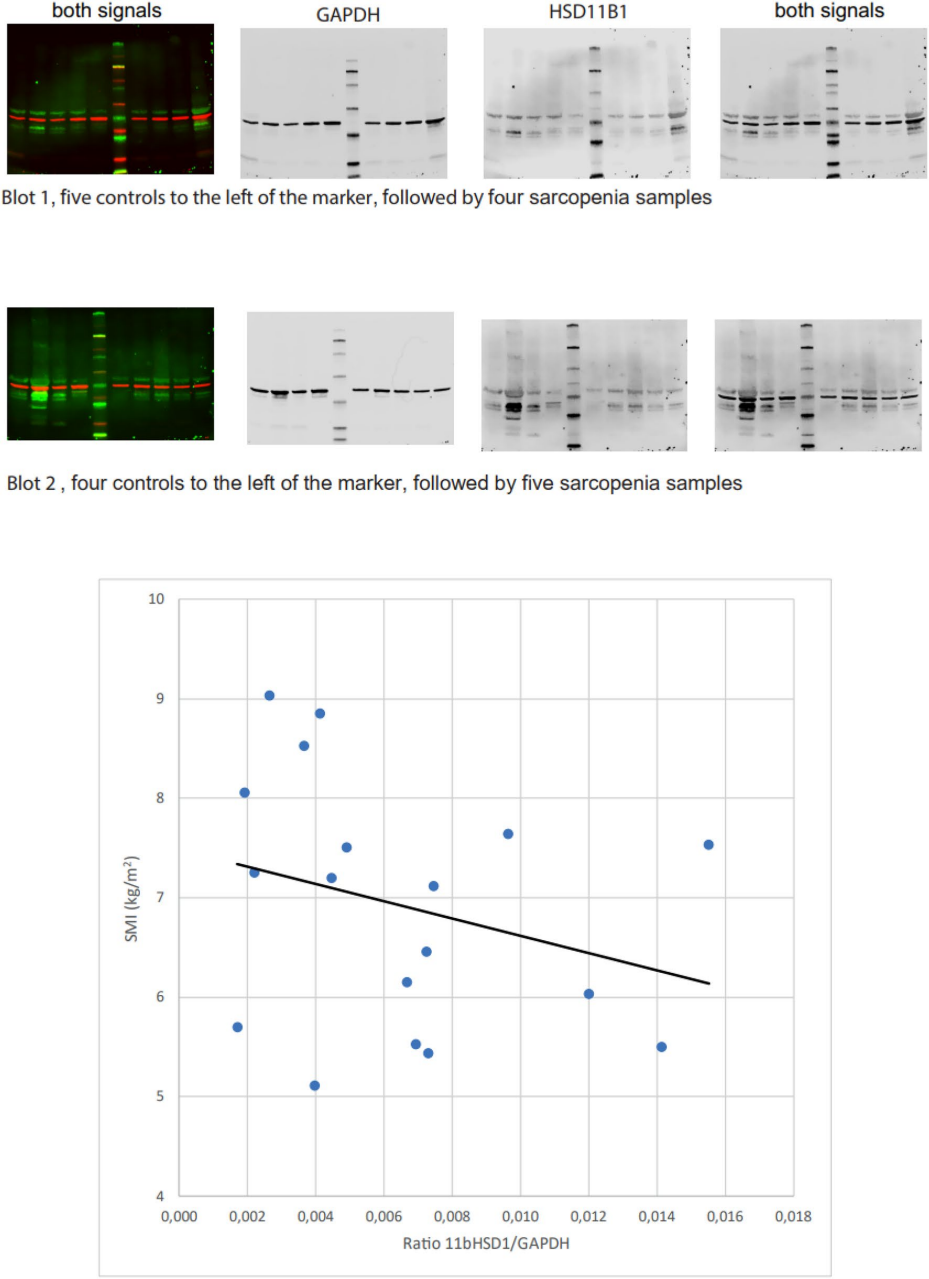
Correlation between SMI and HSD11B1 in Western blot: Pearson’s correlation between SMI (kg/m^2^) and HSD11B1 in a subgroup of nice control and nine sarcopenic samples. Abbreviations: *SMI* Skeletal muscle index, *HSD11B1* Hydroxysteroid 11-Beta-Dehydrogenase 1

Figure 4 displays the Western blot results of HSD11B1 for a mixed group comprising nine sarcopenic and nine control samples, revealing a negative correlation between SMI and HSD11B1 on protein level.

## Discussion

This study aimed to investigate the role of GCs and *HSD11B1* as key regulators of GC action in human sarcopenic muscle cells. The main finding of our study was the negative correlation between *HSD11B1* gene expression and SMI in the sarcopenic group (Fig. 2a), indicating that higher muscle mass is associated with lower expression of *HSD11*B1. Additionally, this correlation was further confirmed at the protein level, as demonstrated in Fig. 4. To the best of our knowledge, this is the first report investigating gene expression of HSD11B1 in a defined sarcopenic group. Reduced expression of HSD11B1 activity can decrease cortisol levels in skeletal muscle and help counteract muscle loss [16]. In mice, selective removal of HSD11B1 prevented skeletal myopathy by reducing the availability of GCs [23]. Low handgrip strength decreased the mass of type-2 fibers, and the increased expression of atrogenes was largely prevented in *HSD11B1* knockout mice treated with GCs [23]. In human skeletal muscle, increased *HSD11B1* mRNA was associated with lower muscle strength in aging men and women [24]. Another cross-sectional study found the same association between muscle strength and *HSD11B1* and discovered sex-specific differences in *HSD11B1* expression in healthy aging patients [25]. In women over 60 years, *HSD11B1* was higher compared to those aged between 20 and 40 years, whereas no significant differences were observed in men [25]. In our study, we could not find any sex-specific differences. This might be due to our cohort's gender composition, including more women than men. Additionally, our investigation focused on muscle tissue samples from older individuals, and we did not compare these findings to muscle tissue samples obtained from a younger control group.

Inhibiting *HSD11B1* can prevent protein degradation caused by GCs in murine myotubes and primary human myoblasts [15, 26]. These findings suggest that locally produced GCs by *HSD11B1* may cause catabolic effects in skeletal muscle via defined protein degradation pathways. Therefore, *HSD11B1* could be a promising target for therapeutic treatments [27]. As indicated, *HSD11B1* expression profiles were already shown in humans for aging but not for sarcopenic muscle. It should be noted that the correlation for HSD11B1 was only observed in the sarcopenic group. The difference in correlation patterns between the sarcopenic and control groups may be due to an alternative molecular signature of muscle loss and atrophy in sarcopenia, compared to a “healthy” muscle tissue situation, as seen in our control group. Elements like muscle remodeling by mesangioblasts and satellite cells must be considered here. This results in distinct patterns of gene expression correlations in the two groups.

In the control group, handgrip strength and SMI positively correlated with *NR3C1* (Table 2, Fig. 2b). The gene encoding the GC receptor, *NR3C1*, can be up- or downregulated in response to cortisol, depending on the context and duration of cortisol exposure [28]. On one hand, high cortisol levels can stimulate the upregulation of *NR3C1*, increasing the number of GC receptors available in cells to bind and respond to cortisol. This can enhance the sensitivity of cells to cortisol and promote a variety of physiological responses, such as the suppression of inflammation and immune function [28]. On the other hand, prolonged exposure to high levels of cortisol, such as may occur with aging, chronic stress, or inflammation, can lead to the downregulation of *NR3C1* [29]. This is considered a protective mechanism to prevent excessive activation of the GC signaling pathway [29]. In our control group, higher expression levels of *NR3C1* were associated with a more favorable SMI, which was not found in the sarcopenic group. This may indicate that this regulation step gets lost in sarcopenic muscle.

The study's second finding was that SMI (Fig. 1a), a marker for muscle mass, and type-2 fiber diameter (Fig. 1b) showed a significant negative correlation with cortisone serum concentrations. This indicates that muscle metabolism measured in this study was not a result of the acute hip fracture situation but rather based on chronic changes, as muscle mass and fiber size are not affected in the short term. Furthermore, this finding suggests that type-2 fiber diameter, a known marker of sarcopenia [18, 30, 31], is associated with cortisone levels, indicating a possible pathomechanism of type-2 fiber diameter loss.

Interestingly, we could only show this association for cortisone but not cortisol. Several reasons might explain this effect. Firstly, serum cortisol concentrations may not accurately reflect local skeletal muscle hormone concentrations [32]. Secondly, the timing of serum cortisol measurements may have been less optimal as they were taken three days postoperatively and not consistently taken in the early morning. Therefore, it is possible that the sampling timing in our study was more sensitive to changes in cortisone levels than cortisol levels. Studies published before the year 2000 suggested that serum cortisol during major surgery rises rapidly but usually returns to baseline values within 24–48 h [33]. However, a recent meta-analysis showed that mean cortisol levels could remain higher than baseline measurements for a longer duration, up to seven postoperative days [34]. It is important to note that most of the selected studies in this meta-analysis did not measure cortisol levels using mass spectrometry [34]. Like our study, Kilgour et al. did not observe any correlation between serum cortisol levels, total urinary GC, and muscle strength or size in a group of healthy patients with a mean age of 80 [24]. Overall, the exact reason for our study's lack of correlation between cortisol levels and sarcopenic markers is unclear and may require further investigation using GC daily or salivary cortisol profiles.

In line with previous studies, our study showed a positive correlation between the atrogene markers *FBXO32* and *TRIM63* and the gene expression of *NR3C1* in skeletal muscle in sarcopenic patients and controls (Figs. 3 a/b) [35–37]. FBXO32 and TRIM63 are E3 ubiquitin ligases that play a crucial role in ubiquitin–proteasome degradation, a major mechanism for breaking down and eliminating unwanted or damaged proteins in cells [5, 6]. In skeletal muscle, FBXO32 and TRIM63 are predominantly expressed during muscle atrophy and are involved in the targeted degradation of specific proteins by the proteasome [5, 6]. Studies have demonstrated that GCs, which bind to NR3C1, can stimulate the expression of FBXO32 and TRIM63 in skeletal muscle cells, leading to increased muscle protein breakdown [35–37]. Our results suggest a relationship among NR3C1, FBXO32, and TRIM63 in skeletal muscle, with NR3C1 potentially playing a role in regulating muscle protein breakdown through the induction of *FBXO32* and *TRIM63* expression.

One of the study's strengths is using muscle biopsies for assessing muscle tissue. Another strength is the standardized assessment of sarcopenia by the EWGSOP2 criteria.

However, this study also has limitations. One area for improvement is the rather small sample size, which may limit the generalizability of the findings. This study only included patients who had undergone hip fracture surgery, and therefore, the results may not apply to the wider population of older adults. Another area of improvement is the cross-sectional study design which cannot prove causality.

## Conclusion

In conclusion, our findings suggest that HSD11B1 might play a pathogenetic role in sarcopenic muscle. Therefore, interventions targeting GCs may be effective in treating sarcopenia. Further studies should investigate the functional consequences of gene expression changes and evaluate the efficacy of GC pathway interventions in preventing or treating sarcopenia.

## Data Availability

The data that support the findings of this study are not openly available due to reasons of sensitivity and are available from the corresponding author upon reasonable request. Data are located in controlled access data storage at Klinikum LMU.
